# miR-224 aggravates cancer-associated fibroblast-induced progression of non-small cell lung cancer by modulating a positive loop of the SIRT3/AMPK/mTOR/HIF-1α axis

**DOI:** 10.18632/aging.202803

**Published:** 2021-04-04

**Authors:** Juan Zhang, Lan Han, Jing Yu, Hui Li, Qingfeng Li

**Affiliations:** 1 Department of Oncology, Xiangyang Central Hospital, Affiliated Hospital of Hubei University of Arts and Science, Xiangyang 441021, Hubei, China

**Keywords:** cancer-associated fibroblasts, non-small cell lung cancer, miR-224, sirtuin 3, hypoxia-inducible factor-1α

## Abstract

Objectives: Cancer-associated fibroblast (CAF) is among the most important tumor-host microenvironment components by affecting tumor progression. This study explored the role of miR-224 in CAF-induced non-small cell lung cancer (NSCLC).

Materials and methods: A CAF-NSCLC cell co-culture model was established, and the miR-224 expression in CAF was detected by reverse transcription-polymerase chain reaction (RT-PCR). Gain- and loss- of experiments of miR-224 were implemented to verify the effects of CAF on NSCLC cell proliferation, invasion, and epithelial-mesenchymal transition (EMT), and endothelial cell (EC) angiogenesis. Overexpressing genetic or pharmacological interventions were performed to explore the potential mechanisms of Sirtuins 3/AMP-activated protein kinase/mammalian target of rapamycin/hypoxia-inducible factor-1α (SIRT3/AMPK/mTOR/HIF-1α).

Results: CAF enhanced the malignant phenotype of NSCLC cells and induced EC angiogenesis. miR-224 was significantly altered in CAFs. miR-224 up-regulation exacerbated NSCLC development mediated by CAFs, while miR-224 inhibition mostly reversed CAF-induced effects. Mechanistically, miR-224 targeted the 3’-untranslated regions (UTR) of SIRT3 mRNA, thereby inhibiting SIRT3/AMPK and activating mTOR/HIF-1α. Forced overexpression of SIRT3 up-regulated AMPK and inactivated mTOR/HIF-1α, while inhibiting HIF-1α markedly up-regulated SIRT3/AMPK and reduced mTOR phosphorylation. Interestingly, both Sirt1 overexpression and HIF-1α inhibition repressed miR-224 levels and miR-224-mediated promotive effects in NSCLC.

Conclusion: The miR-224-SIRT3/AMPK/mTOR/HIF-1α axis formed a positive feedback loop in modulating CAF-induced carcinogenic effects on NSCLC.

## INTRODUCTION

Non-small cell lung cancer (NSCLC), ranking the most common cancer worldwide, has been recognized as the leading cause of death [1–2]. The vast majority of NSCLC patients are diagnosed at an advanced stage because early diagnosis is not possible without clear symptoms [3]. Current standard treatment for advanced NSCLC includes surgery plus chemo- and/or radiotherapy [4]. Despite the continuous development of new treatment methods (such as targeted therapy, immunotherapy, etc.), NSCLC cells' resistance to therapeutic agents has gradually increased in recent years, resulting in relatively poor therapeutic effects [5]. Therefore, it is urgent to study the potential biological mechanism in the development of NSCLC to prevent and treat the disease.

Cancer-associated fibroblasts (CAFs), which are activated by tumors, are important stromal cell components in the tumor microenvironment of solid tumors and play a crucial role in the occurrence and development of various malignant tumors, including NSCLC [6]. CAFs boost the invasion ability of NSCLC and promote the formation of metastasis through paracrine [7]. The secretion of inflammatory cytokines by CAFs is an important mechanism supporting tumor cells. For instance, interleukin-6 (IL6) produced by CAF can enhance the Signal transducer and activator of transcription 3 (STAT3) pathway activation, thus accelerating the proliferation of breast cancer cells [8]. Hepatocyte growth factor (HGF) released by CAF promotes proliferation and chemotherapy resistance of ovarian cancer (OC) cells by regulating the c-Met/ phosphatidylinositol 3-kinase (PI3K) /Akt and glucose related protein 78(GRP78) signaling pathways [9]. These studies indicate the importance of CAFs in the tumor microenvironment. However, the molecular mechanism regulating the crosstalk between CAF and NSCLC needs further investigation.

Noncoding RNAs do not have the ability to encode proteins. In recent years, a mounting number of studies have found that microRNAs (miRNAs) have significant effects on regulating tumor progression. miRNAs contain 19-23 nucleotides. Aberrantly-expressed miRNAs are considered to be promising biomarkers for the development and progression of human diseases [10]. Multiple miRNAs are abnormally expressed and play important regulatory roles in NSCLC [11]. For example, miR-1305 targets urine double minute 2 (MDM2) to regulate the progression of NSCLC [12]. miR-224, one member of miRNAs, has been identified as a biomarker that predicts the treatment response to chemotherapy in diffuse large B-cell lymphoma (DLBCL) patients [13], and also as an oncogene in NSCLC by targeting homeobox protein hox-d10 (HOXD10) [14]. However, the role of miR-224 in the CAF-NSCLC microenvironment remains to be further studied.

In this study, we found that CAFs apparently accelerated the proliferative, invasive abilities as well as the tendency of epithelial-mesenchymal transition (EMT) of NSCLC cells. By using a commercial miRNA microarray for high-throughput screening differential expression miRNAs in CAFs, we found that miR-224 was significantly up-regulated in CAFs and NSCLC cells when they were co-cultured. Functionally, the up-regulation of miR-224 in CAF could further promote the proliferation, metastasis and EMT of NSCLC cells. Moreover, the Sirtuins 3 (SIRT3) and AMP-activated protein kinase (AMPK) were markedly inhibited following miR-224 up-regulation. In contrast, the mammalian/mechanistic target of rapamycin (mTOR)/hypoxia-inducible factor-1α (HIF-1α) signaling pathway was significantly relieved by miR-224. Thus, we supposed there is a miR-224-SIRT3-AMPK-mTOR-HIF-1α axis involved in the CAF-NSCLC microenvironment. Overall, this study aims to probe the role and mechanism of miR-224 in the progression of NSCLC, which will provide a theoretical basis for elucidating the molecular mechanism of NSCLC and provide potential treatment options for NSCLC patients in the future.

## RESULTS

### CAF induced enhanced malignant phenotype of NSCLC cells

**Figure 1:**
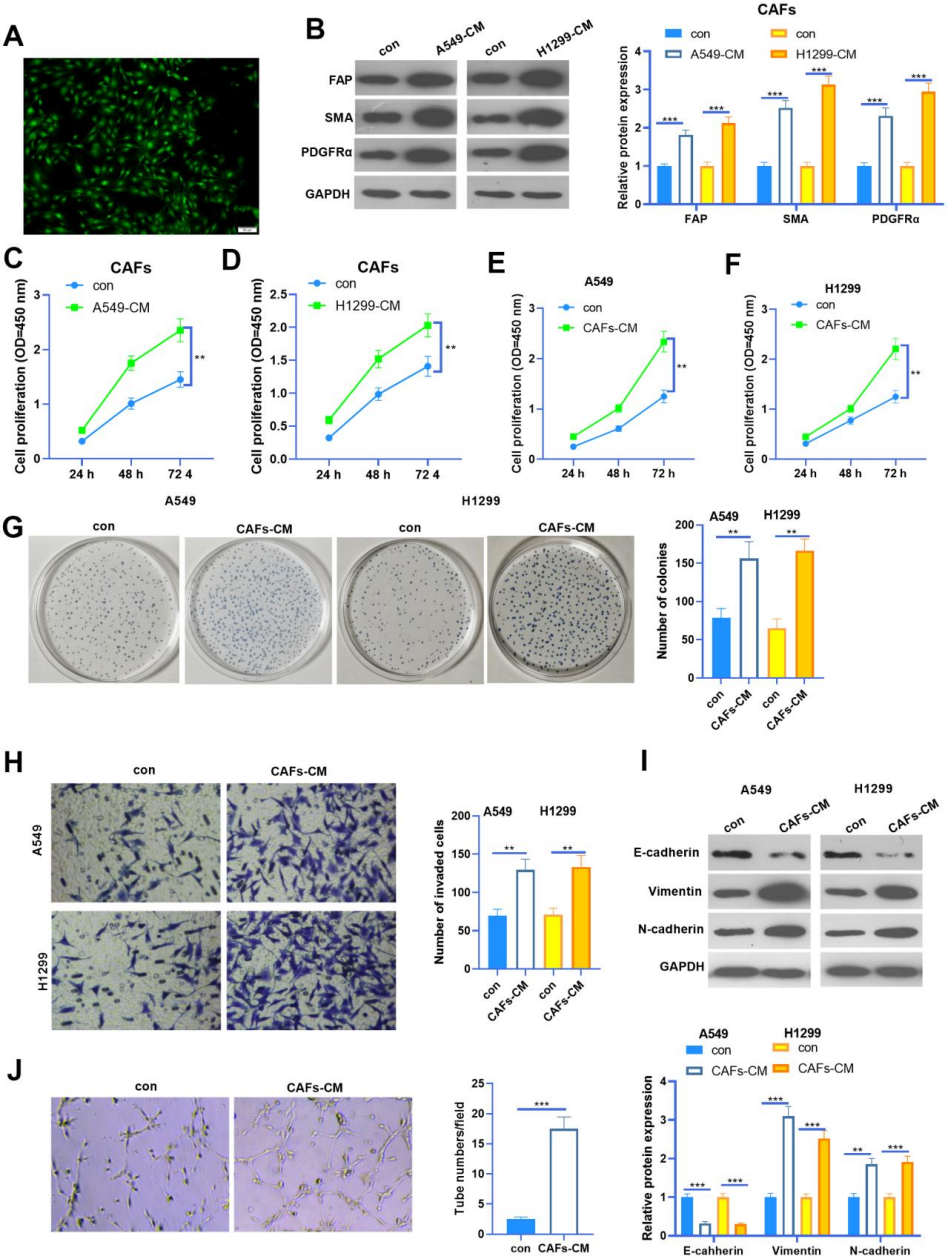
**The interaction between CAF and NSCLC cells.** (**A**) Cellular immunofluorescence (IF) was used to identify CAFs (labeled by SMA). CAFs were co-cultured with NSCLC cell lines (A549 and H1299) or HUVECs for 24 h using Transwell chambers. (**B**) Western blot was performed to detect the CAF markers, including FAP, SMA and PDGFRα in CAFs. (**C**, **D**) CAFs were co-cultured with NSCLC cell lines (A549 and H1299) for different time points (24h, 48h, 72h), and the proliferation of CAFs was determined by the CCK8 assay. (**E**, **F**) CAFs were co-cultured with NSCLC cell lines (A549 and H1299) for different time points (24h, 48h, 72h), and the proliferation of NSCLC cells was determined by the CCK8 assay. (**G**) Colony formation assay was adopted to evaluate the cell colony of NSCLC cells co-cultured with CAFs for 24h, the colonic number was calculated 14 days after incubation. (**H**) Transwell assay was employed to test the invasive ability of NSCLC cells co-cultured with CAFs for 24h. (**I**) EMT markers including E-cadherin, Vimentin, and N-cadherin of NSCLC cells co-cultured with CAFs for 24h were determined by western blot. (**J**) Tube formation assay was implemented to detect the tube formation ability of HUVECs co-cultured with CAFs for 24h. ** represents *P*<0.01, *** represents *P*<0.001. N=3.

We isolated CAFs from NSCLC tissues. The CAFs were identified by their specific markers (including FAP, SMA, and PDGFRα) (Figure 1A, 1B). To figure out the interactions between CAFs and NSCLC cells, we constructed a co-culture model using Transwell chambers. We found that FAP, SMA and PDGFRα profiles in CAFs were significantly enhanced when co-cultured with NSCLC cells (Figure 1B). Next, the cell counting kit-8 (CCK8) assay was used to detect cell proliferation of CAFs and NSCLC cells. It was found that the proliferative abilities of CAFs were potently facilitated when co-cultured with NSCLC cells (Figure 1C, 1D). Interestingly, the proliferation and colony formation of NSCLC cells were also promoted by CAFs (Figure 1E–1G). Next, we used the Transwell assay to test the invasive ability of NSCLC cells. As a result, the invasion of NSCLC cells increased with CAFs co-culturing (Figure 1H). EMT of NSCLC cells was also determined by western blot. The results showed that N-cadherin and Vimentin in NSCLC cells were enhanced when they were co-cultured with CAFs, while E-cadherin was significantly repressed by CAFs (Figure 1I). Furthermore, CAFs were co-cultured with HUVECs, and the angiogenesis of the latter was examined by the tube formation assay (Figure 1J). The results suggested that the tube formation ability of HUVECs was markedly boosted by CAFs. Collectively, CAFs aggravated the malignant behaviors of NSCLC cells and angiogenesis of HUVECs.

### miR-224 was up-regulated in CAFs

**Figure 2:**
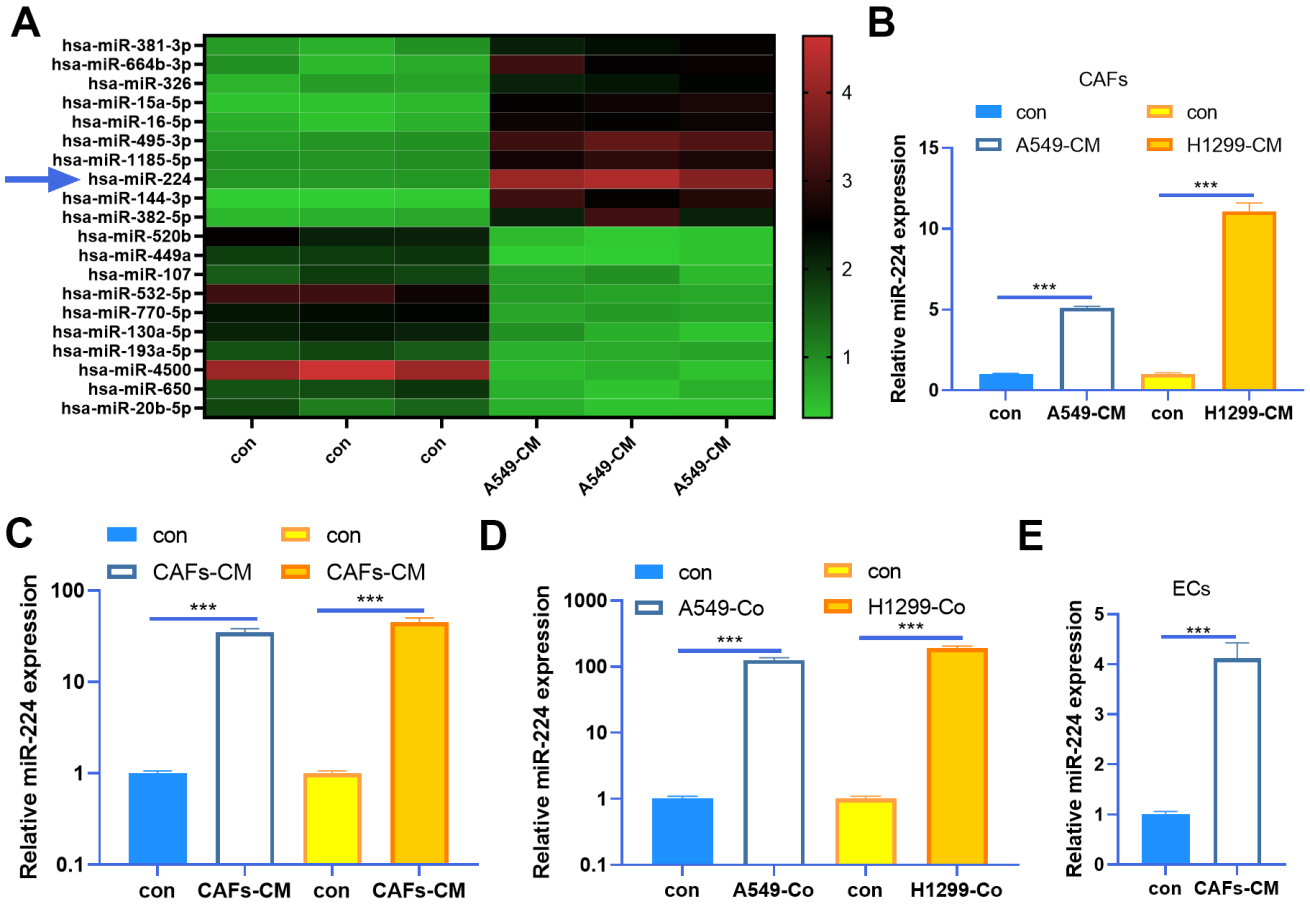
**miR-224 was up-regulated in CAFs and NSCLC cells.** (**A**) CAFs were co-cultured with NSCLC cell lines (A549) for 24 h using Transwell chambers, and a miRNA array was carried out to determine the expression of miRNAs in CAFs. Top ten miRNAs, which were significantly up-regulated or down-regulated, were shown. (**B**, **C**) CAFs were co-cultured with NSCLC cell lines (A549 and H1299) for 24 h. We determined miR-224 expression in both CAFs and NSCLC cells by RT-PCR. (**D**) The level of miR-224 in the co-culture medium of CAFs-NSCLCs was evaluated by RT-PCR. (**E**) miR-224 in HUVECs co-cultured with CAFs was detected by RT-PCR. *** represents P<0.001. N=3.

As miRNAs play a prominent role in tumor progression, we are also curious about the miRNA changes in the CAFs-NSCLC microenvironment. Our data of miRNA arrays showed that miR-224 was significantly up-regulated in CAFs co-cultured with NSCLC cells (Figure 2A). Next, we determined miR-224 expression in both CAFs and NSCLC cells. The results indicated that miR-224 was not only up-regulated in CAFs co-cultured with A549 and H1299 cells, but also was enhanced in the two NSCLC cells when co-cultured with CAFs (Figure 2B, 2C). Meanwhile, we tested miR-224 in the co-culture medium of CAFs-NSCLCs, and it was found that the miR-224 level was significantly increased (Figure 2D). What’s more, we also found that miR-224 was up-regulated in HUVECs when co-cultured with CAFs (Figure 2E). Therefore, we believed that miR-224 plays a role in the CAF-mediated oncogenic effect.

### Overexpression of miR-224 accelerates CAFs-induced promotive effect on NSCLC cells

**Figure 3:**
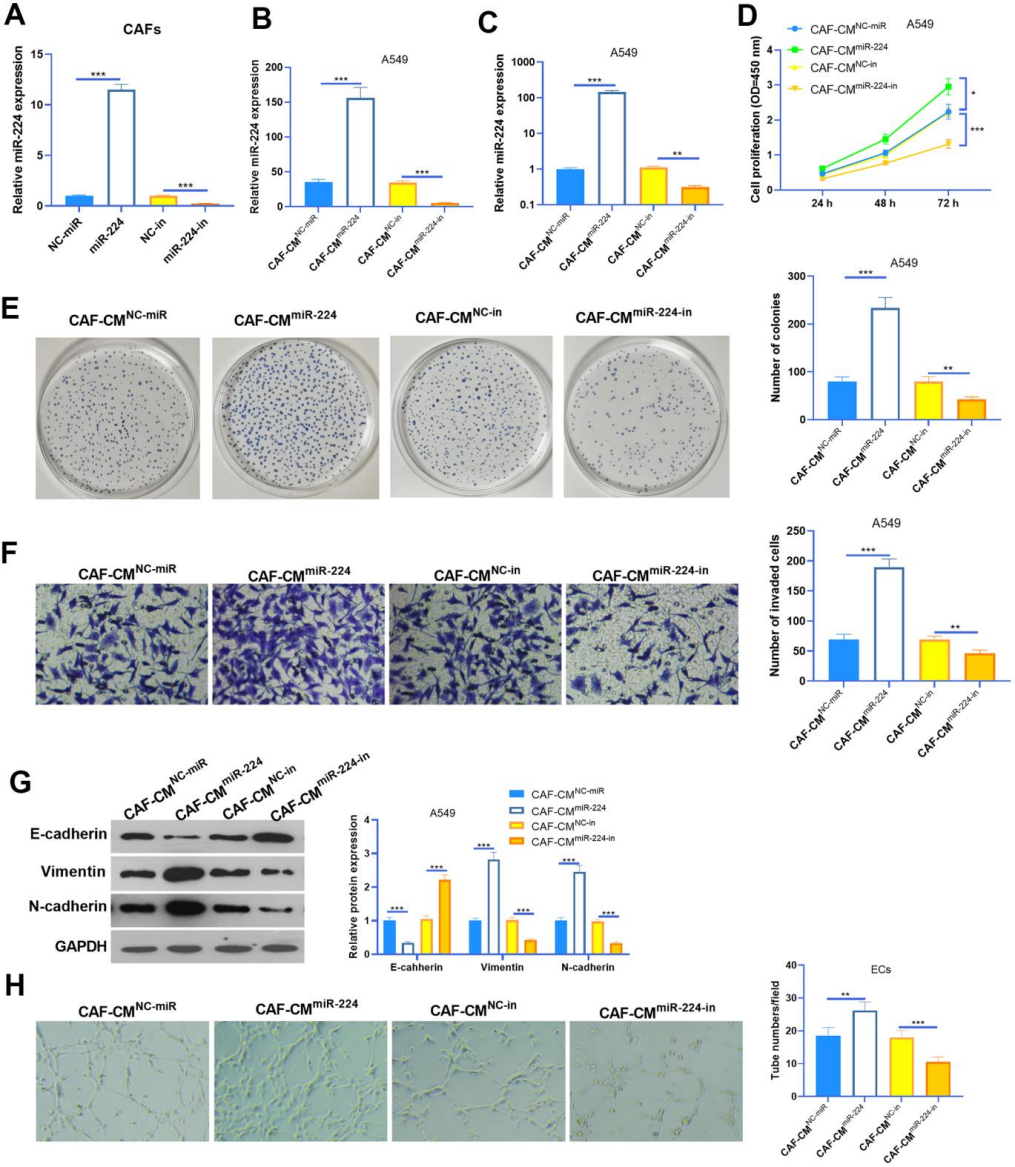
**Effects of miR-224 in CAFs-induced promotive effect on NSCLC cells.** (**A**, **B**) CAFs were transfected with miR-224 mimics or inhibitors for 24 hours, and the level of miR-224 in CAFs (**A**) and the co-culture medium of CAFs-NSCLCs (**B**) was detected by RT-PCR. (**C**) The level of miR-224 in NSCLC cells co-culture with CAFs for 24 hours were detected by RT-PCR. (**D**) The proliferation of NSCLC cells co-culture with CAFs for 24 hours was determined by the CCK8 assay. (**E**) Colony formation assay was used to evaluate the cell colony of NSCLC cells co-culture with CAFs for 24 hours. (**F**) Transwell assay was used to test the invasive ability of NSCLC cells co-culture with CAFs for 24 hours. (**G**) EMT markers including E-cadherin, Vimentin, and N-cadherin of NSCLC cells co-culture with CAFs for 24 hours were determined by western blot. (**H**) Tube formation assay was conducted to monitor the tube formation ability of HUVECs co-culture with CAFs for 24 hours. *, ** and *** represents P<0.05, P<0.01 and P<0.001, respectively. N=3.

We transfected CAFs with miR-224 mimics and inhibitors to modulate miR-224 levels in CAFs (Figure 3A). The results of RT-PCR also showed that the miR-224 level was intervened in the conditioned medium of CAFs (CAFs-CM) (Figure 3B). Next, A549 cells were co-cultured with CAFs with up- or down-regulated miR-224. The results of the CCK8 assay and colony formation assay showed that CAF-CM^miR-224^ significantly accelerated the proliferation of A549 cells (compared with CAF-CM^NC-miR^ treatment), while CAF-CM^miR-224-in^ attenuated the proliferation of A549 cells (compared with CAF-CM^NC-in^ treatment) (Figure 3D, 3E). Next, the invasion and EMT of A549 cells were detected. The results indicated that SOOV-3 cell invasion and EMT were enhanced with CAF-CM^miR-224^ treatment (vs. CAF-CM^NC-miR^), but they were attenuated under CAF-CM^miR-224-in^ treatment (vs. CAF-CM^NC-in^) (Figure 3F, 3G). Furthermore, we detected the tube formation ability of HUVECs by the tube formation assay, which showed that CAF-CM^miR-224^ significantly aggravated HUVEC angiogenesis, while CAF-CM^miR-224-in^ had the opposite effects (Figure 3H). Therefore, we believed that miR-224 is involved in CAF-mediated effects in NSCLC cells and ECs.

### miR-224 promoted CAF-mediated oncogenic effects *in vivo*

**Figure 4:**
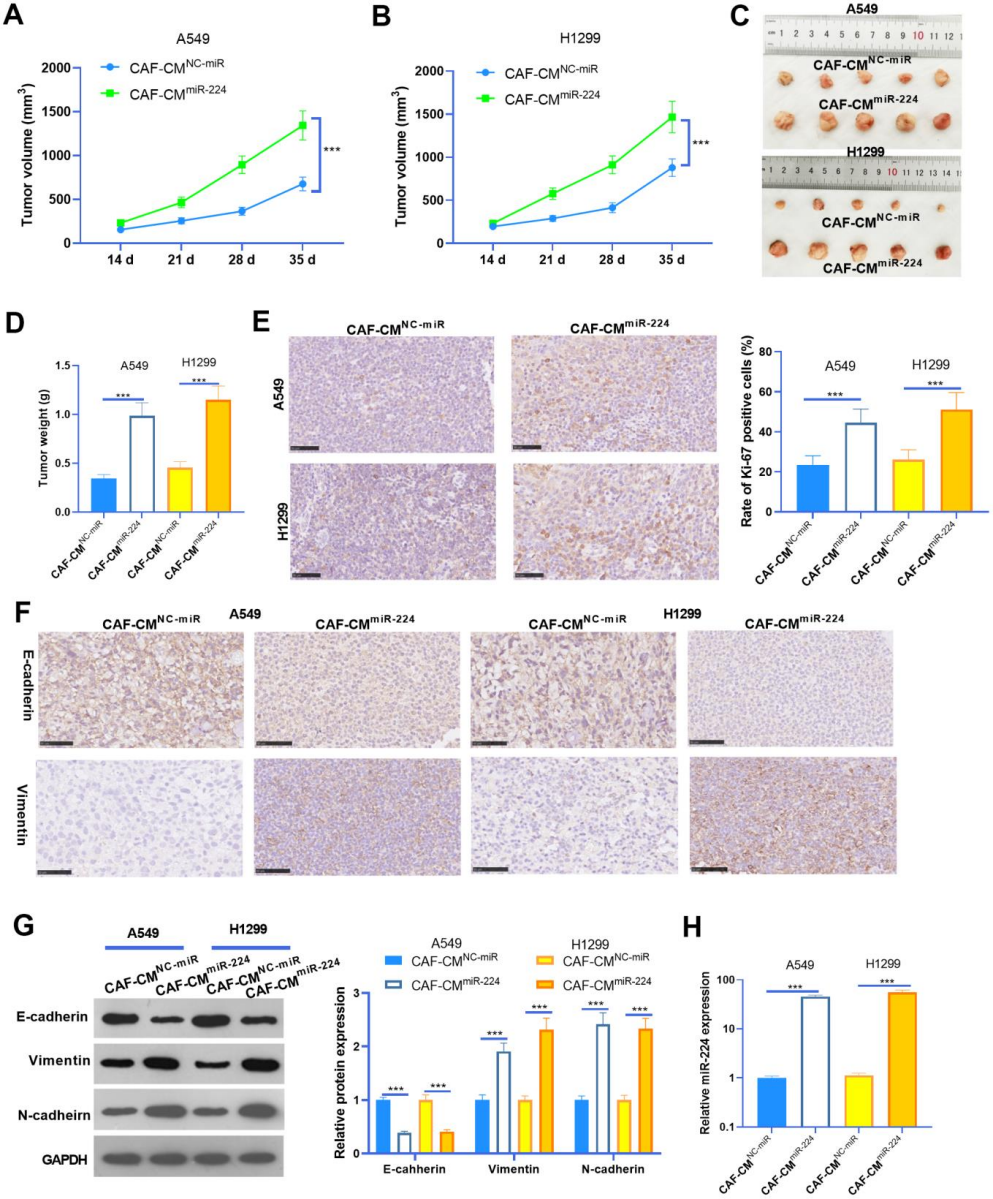
**miR-224 promoted CAF-mediated oncogenic effects *in vivo*.** A549 and H1299 treated with CAF-CMmiR-224 were used for the xenograft tumor experiment in nude mice. (**A**, **B**) Tumor volumes within five weeks of incubation. (**C**, **D**) Tumor images and weight at the fifth week. (**E**) IHC was performed to detect the proliferation (marked by Ki-67) of NSCLC cells, and the rate of Ki-67 positive cells was counted. (**F**) IHC was performed to detect E-cadherin and Vimentin in the tumor tissues. (**G**) The EMT markers of E-cadherin, Vimentin, and N-cadherin in the formed tumor tissues were detected by western blot. (**H**) RT-PCR was implemented to detect miR-224 expression in the tumors. *** represents P<0.001. N=5.

To further probe the role of miR-224 in CAF-induced effects on NSCLC progression, we performed *in vivo* experiments. NSCLC cells A549 and H1299 treated with CAF-CM^miR-224^ were used for the xenograft tumor experiment in nude mice. Consistent with the *in vitro* experiments, the growth of A549 and H1299 cells treated with CAF-CM^miR-224^ were remarkably accelerated (Figure 4A–4D). In addition, we performed IHC to detect the proliferation (marked by Ki-67) of NSCLC cells. As a result, Ki-67 positive cells in the CAF-CM^miR-224^ group significantly increased in comparison to those in the CAF-CM^NC-miR^ group (Figure 4E). Furthermore, the EMT markers in the formed tumor tissues were detected by IHC and western blot. As a result, E-cadherin expression was significantly reduced, while Vimentin and N-cadherin levels were markedly enhanced in the CAF-CM^miR-224^ group compared with the CAF-CM^NC-miR^ group (Figure 4F, 4G). Furthermore, the results of RT-PCR also identified significant-increased miR-224 level in the CAF-CM^miR-224^ group compared with the CAF-CM^NC-miR^ group (Figure 4H). Collectively, the above data proved that miR-224 promoted CAF-induced carcinogenic effects.

### miR-224 modulated the SIRT3-AMPK-mTOR-HIF-1α axis by directly targeting SIRT3

**Figure 5:**
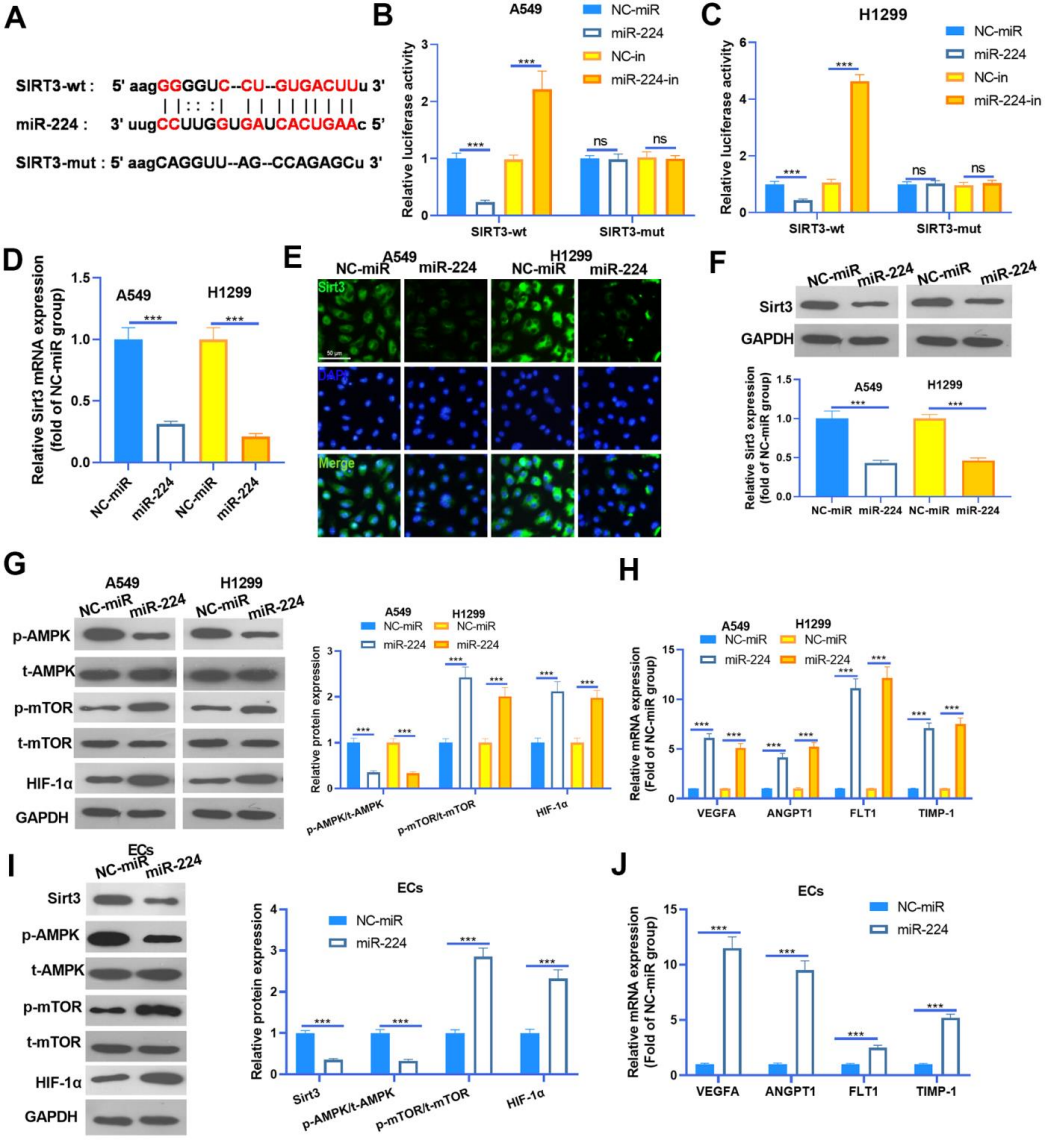
**miR-224 modulated the SIRT3-AMPK-mTOR-HIF-1α axis by directly targeting SIRT3.** (**A**) SIRT3 mRNA contained a binding site with miR-224 as predicted by Starbase (https://web.archive.org/web/20110222111721/http://starbase.sysu.edu.cn/). (**B**, **C**) Dual-luciferase reporter assay was carried out in the two NSCLC cells (A549 and H1299). (**D**) NSCLC cells (A549 and H1299) were transfected with miR-224 mimics and NC-miR, then the mRNA level of Sirt3 in the NSCLC cells was detected by RT-PCR. E-F. Cellular IF (**E**) and western blot (**F**) were conducted to detect Sirt3 expression in NSCLC cells. (**G**) The expression of AMPK/mTOR/HIF-1α in NSCLC cells was monitored by western blot. (**H**) The mRNA levels of VEGFA, ANGPT1, FLT1 and TIMP1 in NSCLC cells were compared by RT-PCR. (**I**) The expression of SIRT3/AMPK/mTOR/HIF-1α in HUVECs was detected by western blot. (**J**) The mRNA levels of VEGFA, ANGPT1, FLT1 and TIMP1 in HUVECs were determined by RT-PCR. NS represents *P*>0.05, *** represents *P*<0.001. N=3.

Studies have confirmed that miR-224 exerts a promotive effect in the progression of cancers by targeting the 3’ (untranslated region) UTR of mRNA [15]. To investigate the underlying mechanism of miR-224 in NSCLC, we searched the potential targets of miR-224 via Starbase (https://web.archive.org/web/20110222111721/http://starbase.sysu.edu.cn/). We found that the Sirt3 mRNA contained a binding site with miR-224 (Figure 5A). To confirm the interactions between miR-224 and SIRT3, the dual-luciferase reporter assay was carried out. As was shown in Figure 5B, 5C, miR-224 mimics significantly reduced the luciferase activity of NSCLC cells transfected with SIRT3-wt, and miR-224 inhibitors significantly increased the luciferase activity of NSCLC cells transfected with SIRT3-wt. However, miR-224 mimics and inhibitors had no marked difference affecting the luciferase activity of NSCLC cells transfected with Sirt3-mut. Next, RT-PCR, cellular IF and western blot were conducted to detect the Sirt3 expression. As was shown, Sirt3 expression was significantly inhibited by miR-224 mimics both in the mRNA and protein level (Figure 5D–5F). Furthermore, western blot was used to determine AMPK/mTOR/HIF-1α in NSCLC cells and HUVECs. The results showed that AMPK phosphorylation was inhibited when miR-224 was overexpressed, while phosphorylated mTOR and total protein of HIF-1α were down-regulated following miR-224 mimic transfection (Figure 5G, 5I). Meanwhile, the mRNA levels of VEGFA, ANGPT1, FLT1 and TIMP1 were all reduced by miR-224 mimics (Figure 5H, 5J). Thus, the above data proved that miR-224 inhibited the SIRT3/AMPK expression and activated the mTOR/HIF-1α signaling pathway via targeting SIRT3.

### miR-224 overexpressed CAFs modulated the SIRT3-AMPK-mTOR-HIF-1α axis

**Figure 6:**
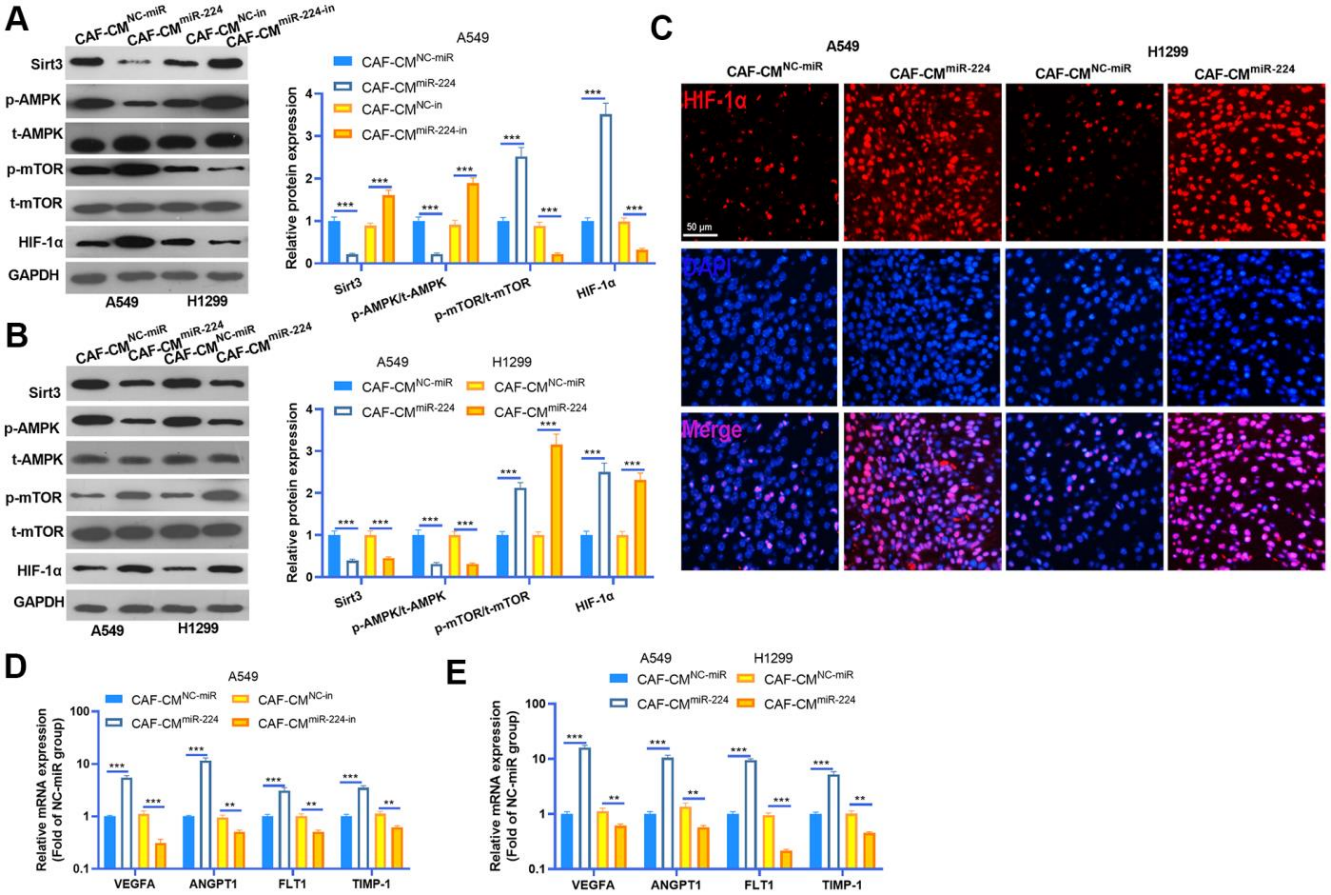
**miR-224 overexpressed CAFs modulated SIRT3-AMPK-mTOR-HIF-1α axis.** (**A**, **B**) Western blot was performed to detected SIRT3-AMPK-mTOR-HIF-1α expression in NSCLC cells and cancer tissues. (**C**) The expression of HIF-1α in the tumors was evaluated by tissue IF. (**D**, **E**) The downstream molecules of HIF-1α, including VEGFA, ANGPT1, FLT1 and TIMP1 were detected by RT-PCR. ** represents *P*<0.01, *** represents *P*<0.001. N=3.

We performed western blot to detected SIRT3-AMPK-mTOR-HIF-1α expression in NSCLC cells and cancer tissues. The results indicated that SIRT3 and AMPK phosphorylation was down-regulated in NSCLC cells treated with CAF-CM^miR-224^, while the mTOR-HIF-1α axis was overexpressed by CAF-CM^miR-224^ (Figure 6A, 6B). However, down-regulation of miR-224 had a reversed effect by increasing SIRT3 and AMPK expression and repressing mTOR/HIF-1α activation (Figure 6A, 6B). Interestingly, we performed tissue IF to detect HIF-1α expression in the cancer tissues. The result revealed that the HIF-1α level in the nucleus was significantly enhanced in the CAF-CM^miR-224^ group (Figure 6C). Furthermore, the downstream molecules of HIF-1α, including VEGFA, ANGPT1, FLT1 and TIMP1 were detected by RT-PCR. It was found that the four molecules were all promoted in the CAF-CM^miR-224^ group (compared with the CAF-CM^NC-miR^ group both *in vitro* and *in vivo*) (Figure 6D, 6E). In contrast, the CAF-CM^miR-224-in^ group had a lower level of VEGFA, ANGPT1, FLT1 and TIMP1 (Figure 6D). Taken together, miR-224 affected CAF mediated effects on NSCLC cells via the SIRT3-AMPK-mTOR-HIF-1α pathway.

### miR-224-SIRT3-AMPK-mTOR-HIF-1α axis formed a feedback loop in NSCLC cells

**Figure 7:**
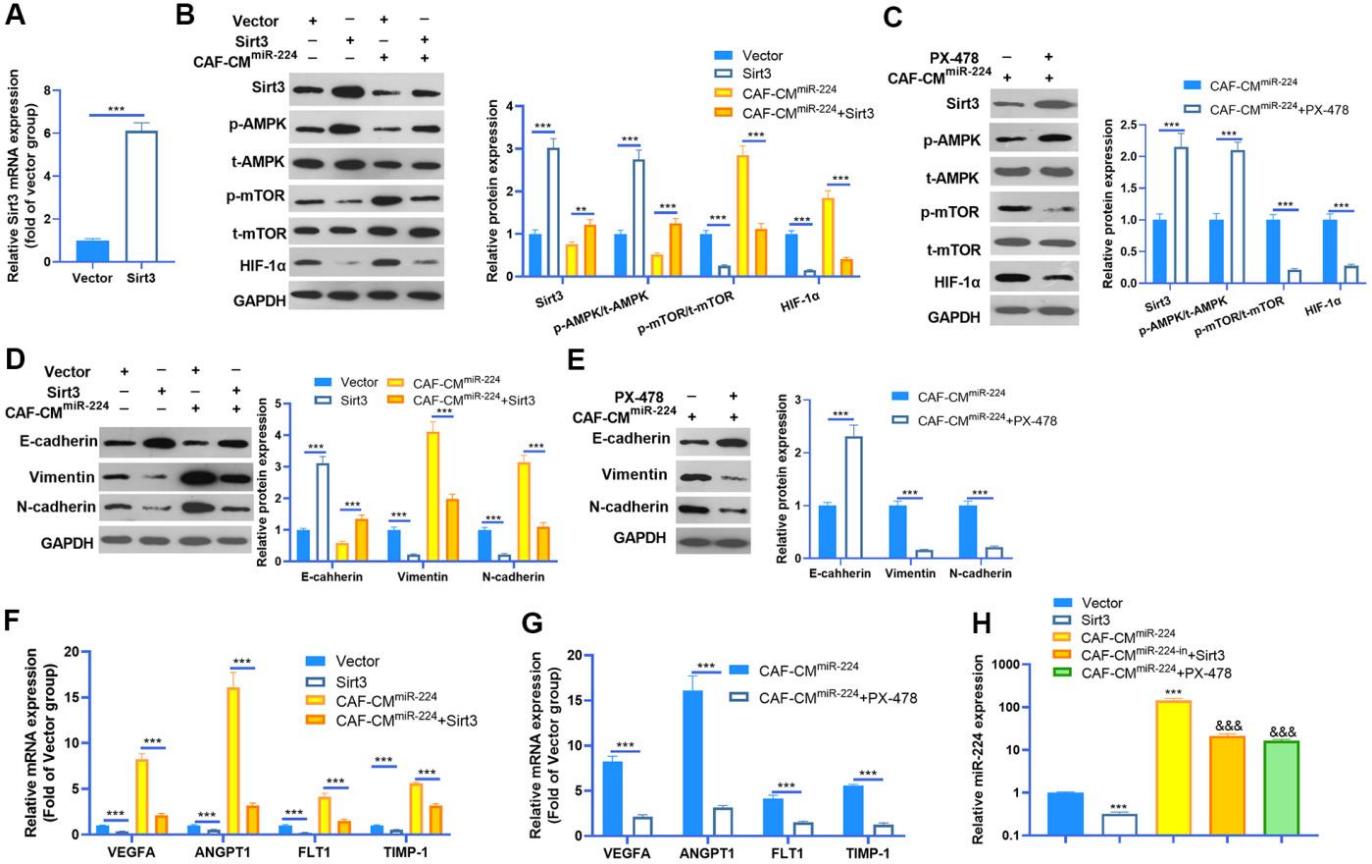
**miR-224-SIRT3-AMPK-mTOR-HIF-1α axis formed a feedback loop in NSCLC cells.** (**A**) The SIRT3 overexpressing model was constructed in A549 cells using SIRT3 overexpression plasmids transfection for 24 hours. The SIRT3 level was detected by RT-PCR. (**B**) A549 cells (normal or SIRT3 overexpression) were treated with CAF-CMmiR-224 for 24 hours. The SIRT3-AMPK-mTOR-HIF-1α expression was detected by western blot. (**C**) A549 cells were treated with CAF-CMmiR-224 and/or HIF-1α inhibitor PX-478 (40 μM) for 18 hours. The SIRT3-AMPK-mTOR-HIF-1α expression was examined by western blot. (**D**, **E**) EMT markers, including E-cadherin, Vimentin, and N-cadherin of NSCLC cells were determined by western blot. (**F**, **G**) The downstream molecules, including VEGFA, ANGPT1, FLT1 and TIMP1 in A549 cells were tested by RT-PCR. *** represents P<0.001. (**H**) The miR-224 level was examined by RT-PCR. *** represents P<0.001 vs. Vector group, &&& represents P<0.001 vs. CAF-CMmiR-224 group. N=3.

**Figure 8:**
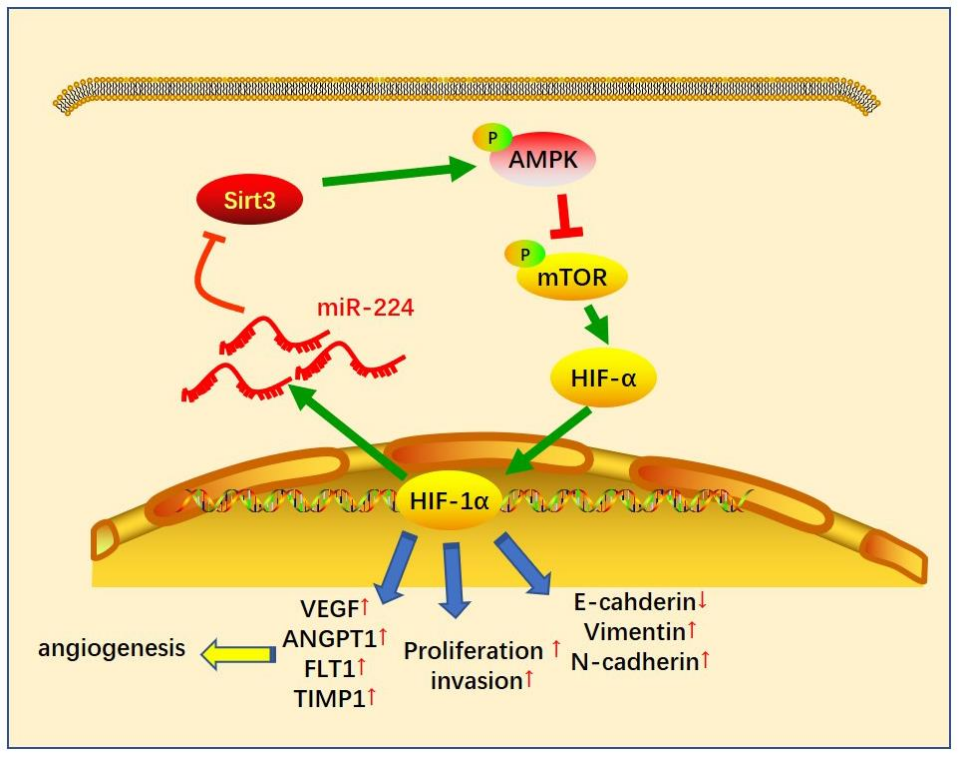
**The sketch map of the regulatory loop of the miR-224-SIRT3-AMPK-mTOR-HIF-1α axis.** miR-224 targeted SIRT3 and inhibited AMPK, followed by up-regulation of mTOR-HIF-1α. A higher level of HIF-1α then enhanced the transcription of miR-224 as well as the other genes in promoting cell growth, angiogenesis and metastasis of NSCLC cells.

Given the significant role of miR-224 in regulating SIRT3-AMPK-mTOR-HIF-1α in NSCLC cells, it was necessary to further confirm the underlying mechanism of the SIRT3-AMPK-mTOR-HIF-1α axis in NSCLC cells. The SIRT3 overexpression model was constructed in A549 cells (Figure 7A), while PX-478 was used to inhibit HIF-1α. We then found that enforced up-regulation of SIRT3 increased AMPK phosphorylation and inactivated the mTOR/HIF-1α pathway (Figure 7B). Noteworthily, up-regulation of SIRT3 partly reversed the CAF-CM^miR-224^-induced SIRT3-AMPK inhibition and mTOR/HIF-1α pathway overactivation (Figure 7B). On the other hand, inhibition of HIF-1α also attenuated CAF-CM^miR-224^-modulated SIRT3-AMPK-mTOR-HIF-1α, namely, enhancing SIRT3/AMPK while repressing the mTOR/HIF-1α pathway (Figure 7C). We determined the EMT of A549 cells. The results indicated that overexpression of SIRT3 or inhibition of HIF-1α restrained the EMT of A549 cells induced by CAF-CM^miR-224^ (Figure 7D, 7E). Furthermore, we tested the downstream molecules of HIF-1α by RT-PCR, which showed that VEGFA, ANGPT1, FLT1 and TIMP1 were all inhibited with SIRT3 overexpression and HIF-1α inhibition (Figure 7F, 7G). The miR-224 level was examined by RT-PCR. Interestingly, the data showed that forced up-regulation of SIRT3 led to a lower level of miR-224, and HIF-1α inhibition also markedly attenuated miR-224 (compared with the CAF-CM^miR-224^ group). Therefore, we believed that there was a regulatory loop of the miR-224-SIRT3-AMPK-mTOR-HIF-1α axis. miR-224 targeted SIRT3 and inhibited AMPK, followed by the up-regulation of mTOR-HIF-1α. The higher level of HIF-1α then enhanced the transcription of miR-224 as well as the other genes in promoting cell growth, angiogenesis, and metastasis (Figure 8).

## DISCUSSION

In this study, we explored the role of miR-224 in regulating CAF-mediated oncogenic effects on NSCLC cells. Our results demonstrated that miR-224 promotes the progress of NSCLC as an oncogene. Relevant mechanism studies have confirmed that miR-224 accelerates CAF-induced NSCLC cell proliferation, EMT and invasion through the SIRT3/AMPK/mTOR/HIF-1α signaling pathway.

The occurrence of NSCLC is not only caused by gene mutations, uncontrolled cell proliferation, and other factors. The tumor microenvironment (TME) also plays an indispensable role in the tumorigenesis and development of NSCLC [16]. Changing the immune microenvironment within the tumor has been regarded as an effective method in the immunotherapy of NSCLC [17]. Within TME, the tumor stroma is composed of a large number of non-tumor cells. Among them, CAFs are the main components in the tumor stroma. Studies have shown that CAFs play a crucial role in malignant progression, drug resistance acquisition, distant metastasis, angiogenesis and recurrence of NSCLC [18, 19]. For instance, interleukin-22 (IL-22) was profoundly elevated in cell cultures of primary CAFs, and CAFs-secreted IL-22 aggravates the proliferation and metastasis of lung cancer cells via the PI3K-Akt-mTOR signaling pathway [20]. In this study, we explored the effects of CAF on the proliferation, EMT, invasion of NSCLC cells and angiogenesis of ECs. Our results indicated that after the co-culture with CAF, the proliferation, EMT, and invasion of NSCLC cells and angiogenesis of ECs were all markedly enhanced. Mechanistically, CAFs can promote the invasion ability of NSCLC and the formation of metastases through the paracrine effect. For example, VEGF [21] and ANGPT1/2/4 [22] produced by CAFs can significantly promote the angiogenesis of tumor cells. Different patterns of cytokine and angiogenic factor expression is found to indicate the progression of NSCLC patients under vandetanib and/or chemotherapy [23]. In this study, we found that CAFs induced a higher level of VEGFA, ANGPT1, FLT1 and TIMP1 in the NSCLC cells, indicating that CAF-mediated NSCLC carcinogenic effects are closely related to these cytokines.

miRNAs play a vital role in regulating the malignant phenotype of tumors [24]. By specifically binding to the 3'UTR of the targeted gene, miRNAs achieve the regulation of gene expression at the post-transcriptional or translational level. For instance, miR-26a is down-regulated in OC and impedes the proliferation, migration and invasion of OC cells by targeting TCF12 [25]. Similarly, miR-331-3p inhibits the proliferation and metastasis of NSCLC cells by targeting ErbB2 and VAV2 through the Rac1/PAK1/β-catenin axis [26]. Here, we performed miRNA arrays to detect the altered miRNAs in CAFs when co-cultured with NSCLC cells. It was revealed that miR-224 is significantly changed. Functionally, CAFs with overexpressed miR-224 have augmented effects on NSCLC progression, which verifies the oncogenic role of miR-224 in NSCLC mediated by CAFs. Actually, increasing studies have suggested that miR-224 can predict the unfavorable outcome of cancers and also aggravate cancer development. For instance, the higher level of miR-224 was associated with poorer survival in hepatocellular carcinoma [27], cervical cancer [28], and other cancers [29]. In addition, miR-224 has prominent roles in affecting the malignant behaviors of cancers, such as promoting proliferation, chemical drug resistance and metastasis [30–33]. Interestingly, miR-224 also accelerates NSCLC development. For example, miR-224 is up-regulated in NSCLC patients and facilitates proliferation and migration of NSCLC cells in part by directly targeting CASP7 [34]. Thus, miR-224 is a promising therapeutic target in NSCLC.

SIRT3, as a member of the sirtuin family, belongs to a mitochondrial NAD^+^-dependent deacetylase and regulates mitochondrial function and biosynthetic pathways [35]. Recently, accumulating studies have revealed that SIRT3 prevents the formation of cancer cells. For example, SIRT3 expression was significantly down-regulated in prostate cancer (PC), and it restrained the EMT and migration of PC cells through inactivating the promoted FOXO3A-mediated Wnt/β-catenin pathway [36]. What’s more, SIRT3 also participates in regulating the proliferation, apoptosis, chemotherapy sensitivity and metastasis of NSCLC [37–39]. Presently, we searched the potential targets of miR-224, aiming at further exploring the underlying mechanisms of miR-224. We testified that miR-224 targeted the 3’-UTR of SIRT3, suggesting that miR-224 functioned its oncogenic effects by negatively regulating SIRT3, a tumor-suppressive gene in NSCLC.

Adenosine monophosphate-activated protein kinase (AMPK), also known as protein kinase AMP-activated catalytic subunit alpha 1 (PRKAA1), is a vital member of the ser/thr protein kinase family [40]. Fundamentally, AMPK is a cellular energy sensor conserved in all eukaryotic cells, and AMPK kinase becomes activated by the increased AMP/ATP ratio in the cellular space [41]. More importantly, AMPK also modulates carcinogenesis and cancer drug resistance as a stress-response molecule with an emphasis on its duplex implication [42]. In NSCLC, metformin enhances the sensitivity of H1975 and PC-9GR cells to osimertinib via inhibiting autophagy dependently through activation of AMPK [43]. Interestingly, another study suggested that SIRT3 overexpression facilitates metformin-induced energy stress and apoptosis in OC cells via activating AMPK [44]. In this study, we found the AMPK phosphorylated level was significantly repressed by CAF-CM^miR-224^ both *in vitro* and *in vivo* experiments. In contrast, forced up-regulation of SIRT3 enhanced AMPK activation, and also rescued CAF-CM^miR-224^-mediated inhibitive effects on AMPK.

Mammalian/mechanistic target of rapamycin (mTOR) is an atypical serine/threonine-protein kinase. The mTOR signaling regulates amino acid, glucose, nucleotide, fatty acid, and lipid metabolism, and then participates in cell growth, survival, exercise and other process adjustments [45]. In malignant tumors, the PI3K/AKT/mTOR pathway plays a critical role in the malignant transformation of human tumors and their subsequent growth, proliferation, and metastasis [46]. This phenomenon indicates that mTOR exerts a great effect on regulating the progression of cancer. On the other hand, activation of HIF-1α is an important mechanism that mediates tumor progression. Activated HIF-1α can up-regulate multiple cytokines such as vascular endothelial growth factor (VEGF), which in turn stimulates the development of new blood vessels to enrich tumor cell growth with oxygen. In addition, HIF-1α can also activate oncogenic growth factors such as transforming growth factor beta3 (TGF-β3) and epidermal growth factor (EGF) through transcription to promote tumor metastasis to distant and oxygenated tissues [47]. In short, HIF-1α activation in tumor cells is one of the mechanisms that guide its adaptation to hypoxia. For example, HIF-1α was increased in NSCLC cells after irradiation treatment and promoted the transcription of CXCR4. Thus, the metastasis and invasiveness of H1299, A549 and H460 cells were significantly enhanced [48]. In this study, we found that the mTOR/HIF-1α signaling pathway was markedly enhanced following CAF-CM^miR-224^ treatment, accompanied by the up-regulation of downstream genes (including VEGFA, ANGPT1, FLT1 and TIMP1) of HIF-1α. As a matter of fact, previous studies have revealed that miR-224 can activate both mTOR and HIF-1α [49, 50]. Therefore, we believe that CAF-CM^miR-224^ regulates the NSCLC development via activating the mTOR/HIF-1α signaling pathway.

It is noteworthy that HIF-1α acts not only as a key response adapted to tumor hypoxia, but also a transcription factor controlling a plethora of target genes that promote physiological changes associated with chemo-/radioresistance [51]. Interestingly, HIF-1α can up-regulate miR-224 and then affect oncogenesis. For example, HIF-1α involves in NK cells-induced PC cell death via increasing miR-224 expression [52]. miR-224 is up-regulated by hypoxia and HIF-1α, thereby inducing gastric cancer (GC) cell growth, migration and invasion [53]. Given the evidence that AMPK represses the mTOR pathway in cancer [54], we supposed there is a regulatory loop of miR-223-SIRT3-AMPK-mTOR-HIF-1α in CAF-mediated effect. Here, we found that SIRT3 overexpression up-regulates AMPK and inactivates the mTOR-HIF-1α pathway, and also attenuates miR-224 expression. Similarly, inhibiting HIF-1α by its specific inhibitor PX-478 enhances SIRT3/AMPK expression and represses mTOR phosphorylation and miR-224 level.

In summary, this study demonstrated *in vitro* and *in vivo* that overexpressed miR-224 in NSCLC-CAF derives the progression of NSCLC via modulating the SIRT3-AMPK-mTOR-HIF-1α signaling pathway. The up-regulated miR-224 forms a positive feedback loop of miR-224-SIRT3-AMPK-mTOR-HIF-1α. Overall, this study explains new molecular mechanisms in the progression of NSCLC and provides a new reference for the diagnosis and treatment of it.

## MATERIALS AND METHODS

### Cell culture and treatment

Human NSCLC cell lines A549, H1299, human umbilical vein endothelial cells (HUVECs) were purchased from the American Type Culture Collection (ATCC). Cells were cultured in the complete RPMI1640 medium containing 10% inactivated newborn bovine serum (FBS, HyClone, Logan, UT, USA) and 1% penicillin/streptomycin. They were cultured at 37° C in an incubator with saturated humidity and 5% CO_2_. The medium was changed every 2 to 3 days. Cells were trypsinized and sub-cultured with 0.25% trypsin during the logarithmic growth phase. To inhibit HIF-1α in A549 cells, SKPV-3 cells were treated with PX-478 (40 μM) (Selleck Chemicals, Houston, TX, USA) for 18 hours [55].

### Isolation of NSCLC CAF

Five cases of cancer tissues were taken from patients diagnosed with NSCLC in the Xiangyang Central Hospital from February to April 2018. The fresh tissues were fully washed with ice PBS within 0.5 h and were separated by aseptic operation of the surgical blade, and then placed in 0.1 mg/ml collagenase for 3 h. After filtration, the red blood cells were lysed, washed with PBS, and cultured with the McCoy's 5A complete medium. Normal fibroblasts were obtained from CAF by gradient digestion, and the cells were continuously cultured in a complete RPMI1640 medium containing 10% inactivated FBS (HyClone, Logan, UT, USA) and 1% penicillin/streptomycin. Cells were trypsinized and sub-cultured with 0.25% trypsin during the logarithmic growth phase. Moreover, the CAF protein markers (including FAP, SMA and PDGFRα) were detected by western blot to identify CAF.

### Cell transfection

After being washed by PBS, NSCLC cells (A549 and H1299) and CAFs were trypsinized (0.25% Trypsin, Beyotime Biotechnology, Wuhan, China) for 2 min and inoculated into 12-well plates with a cell density of 1×10^6^ /mL, achieving a confluence rate of 50-60%. The transfection reagent was diluted in 3 μL/L serum-free medium and incubated at 37° C for 20 min. The miR-224 mimics, miR-224 inhibitors, SIRT3 overexpression plasmids, and their corresponding negative controls, which were designed and integrated by GenePharma (Shanghai, China), were diluted in RPMI1640 medium without serum and incubated for 5 min at room temperature. In the end, it was mixed with the same volume of Lipofectamine 2000 (11668-027, Invitrogen Inc., Carlsbad, CA) and cultured in a 37° C incubator. After 24 h, the serum-free medium was exchanged by the complete medium. After a continuous 48-h culture, the total RNA in the cells was extracted and subjected to RT-PCR to verify the transfection efficiency.

### CCK8 method

2×10^3^ cells from each group were inoculated into 96-well plates. After 24 h of adherent culture, each well was supplemented with 10 μL CCK-8 solution (Beyotime Biotechnology, Wuhan, China). After incubating at 37° C for 4 hours, the absorbance of each well was measured at 450 nm by a microplate reader. Based on this, the changes in cell proliferation of NSCLC cells and CAFs at 24h, 48h and 72h were observed.

### Matrigel tube formation assay

Matrigel (Corning, Lake Franklin, New Jersey, USA) was precoated in a 96-well plate and then got solidified at 37° C for 30 min. HUVECs were collected and resuspended in the serum-free RPMI 1640 medium. Then, 200 *μ*l of cell supernatants were added to each well containing 1×10^4^ HUVECs and incubated at 37° C and 5% CO_2_. Eight hours later, a microscope was used to observe the tube formation, and the honeycomb-like tubular structures were quantified.

### Microarray analysis

CAFs co-cultured with/without NSCLC cells were collected, and the total RNA was extracted using the TRIzol™ reagent (Thermo Fisher Scientific, Waltham, MA, USA). Then the miRNAs expression was analyzed by TaqMan® MicroRNA arrays (384-Well Microfluidic Cards) on the Quantstudio™ 7 Flex real-time PCR instrument (Applied Biosystems®, ThermoFisher Scientific, Waltham, MA, USA). The quantification of RNA was determined by Spectrophotometry and Agilent Bioanalyzer (Agilent Technologies, Santa Clara CA). The differential expression genes were screened by the R programme, bayesian test and fold change.

### Cellular immunofluorescence

NSCLC cells (A549 and H1299) were seeded on glass coverslips and cultured overnight. Then they were fixed with 4% paraformaldehyde, permeabilized with 0.2% Triton X-100, blocked with 5% bovine serum albumin, and incubated with anti-SIRT3 primary antibody (cat. no. sc-99143, 1:3,000; Santa Cruz Biotechnology, Inc.) at 4° C overnight. After being washed by PBS 3 times (5 min each time), the cells were incubated with Fluorescein (FITC)-conjugated Affinipure Goat Anti-Rabbit IgG (H+L) (cat. no. SA00003-2, Proteintech, Shanghai, China) for 1 hour. Finally, 4′,6-diamidino-2-phenylindole (Beyotime Biotechnology, Wuhan, China) was used to stain the cell nuclei. After washing with PBS, the cells were imaged and counted under a fluorescence microscope, and the results were analyzed.

### Immunohistochemical (IHC) and Immunofluorescent (IF) analysis of tissues

The xenograft tumor tissues were fixed with paraformaldehyde and then embedded in paraffin. Next, the 0.5 mm tumor sections were prepared, baked at 60° C for 2 hours, sliced, dried, deparaffinized as well as rehydrated for 10 min in xylene and graded ethanol. Antigen retrieval was implemented by heating (100° C) for 25 min in citrate buffer (10 mM, pH 6.0). The slides were then sealed with goat serum, and incubated with the anti-Ki-67 primary antibody (1:50; cat. no. MA5-14520; Thermo Fisher Scientific, Inc.) and anti-HIF-1α primary antibody (1:100; cat.no. ab179483, Abcam, Cambridge, MA, USA) overnight at 4° C. Then, slides were rinsed twice with PBS, and then incubated with corresponding secondary antibodies. After PBST washing again, the sections incubated with the anti-Ki-67 primary antibody were supplemented with diaminobenzidine. Hematoxylin was used for counterstaining for 30 s, and then the sections were dehydrated, cleared in xylene and treated with neutral gum at last. While for the sections incubated with the anti-HIF-1α primary antibody, they were counterstained by DAPI (1: 2000; Millipore Sigma). After being flushed with PBS, Ki-67 expression was observed under a light microscope, and HIF-1α-immunofluorescent signaling was examined with a fluorescence microscope (Olympus CX23, Tokyo, Japan).

### Transwell assay

A transwell cell invasion experiment was used to detect NSCLC cell invasion. 2×10^4^ NSCLC cells/well were added to the upper chamber of Transwell precoated with Matrigel, and 600 μL of a culture solution containing 20% FBS was added to the lower chamber and cultured at 37° C. After 12 h, the upper chamber cells were removed, fixed with 4% paraformaldehyde, and stained with 0.1% crystal violet (Beyotime Biotechnology, Wuhan, China). After drying, they were photographed and counted.

### RT-PCR

The total RNA was extracted from the cell line and xenograft tumor tissues with the TRIzol reagent (Thermo Fisher Scientific, Waltham, MA, USA). Then the extracted RNA was reversely transcribed into cDNA using the ReverTra Ace qPCR RT Kit (Toyobo, Osaka, Japan). Followed by that, the SYBR Green PCR reagent (Thermo fisher scientific inc, Waltham, MA, USA) and ABI7500FAST Real-Time PCR instrument (ABI Life Technologies, Singapore) were used for quantitative Real-Time PCR. The 2^-ΔΔCt^ method was used to evaluate the expression levels of miR-224 (U6 as the internal control), SIRT3, VEGFA, ANGPT1, FLT1 and TIMP1(GAPDH as an internal control). The primers (Sangon Biotech, Shanghai, China) were shown in Table 1.

**Table 1:** Primers used in this study.

**cDNA**	**Sequences (5′–3′)**
miR-224(F)	CTGGTAGGTAAGTCACTA
miR-224(R)	CAACTGGTGTCGTGGAG
U6 (F)	CTCGCTTCGGCAGCACATA
U6 (R)	AACGATTCACGAATTTGCGT
VEGFA(F)	CCCACTGAGGAGTCCAACAT
VEGFA(R)	TTTCTTGCGCTTTCGTTTTT
ANGPT1F)	GAAGGGAACCGAGCCTATTC
ANGPT1(R)	GGGCACATTTGCACATACAG
FLT1(F)	AGGGGAAGAAATCCTCCAGA
FLT1(R)	TCCTCCGAGCCTGAAAGTTA
TIMP1(F)	AATTCCGACCTCGTCATCAG
TIMP1(R)	TGCAGTTTTCCAGCAATGAG
GAPDH (F)	GAAGGTGAAGGTCGGAGTC
GAPDH (R)	GAAGATGGTGATGGGATTTC

### Colony formation assay

Single-cell suspensions of NSCLC cells were made by trypsin. Then the cells were resuspended by 10 ml of prewarm RPMI 1640 medium and seeded in the Petri dish. Each dish contained 1×10^3^ cells. Then the Petri dish was put in an incubator with a humidified air atmosphere with 5% CO_2_ at 37° C. After a 10-day incubation, the cells were fixed with 4% paraformaldehyde and then dyed with GIMSA (Beyotime Biotechnology, Wuhan, China) for 15-30 min. The number of colonies was observed under a light microscope and counted.

### Western blot

After the cells were processed, we discarded the culture medium, added protein lysate (Roche), and isolated the total protein. 50 μg of total protein was added to a 12% polyacrylamide gel and ran at 100V for 2 h. It was electrically transferred to polyvinylidene fluoride (PVDF) membranes. After being blocked with 5% skimmed milk powder for 1 hour at room temperature, the membranes were washed 3 times with TBST for 10 min each time. Then the membranes were incubated at 4° C overnight with the primary antibodies of anti-SIRT3 (cat. no. sc-99143, 1:3000; Santa Cruz Biotechnology, Inc.), mTOR (1:1000, cat.no. ab2732, Abcam, Cambridge, MA, USA), mTOR (phospho S2448) (1:1000, cat.no. ab109268), AMPK alpha 1 (1:1000, cat. no. ab32047), AMPK alpha 1 (phospho T183) (1:1000, cat.no. ab133448), HIF-1α (1:1000, cat. no. ab51508), FAP (1:1000, cat.no. ab207178), SMA (1:1000, cat.no. ab5831), PDGFRα (1:1000, cat.no. ab203491), E-cadherin (1:1000, cat.no. ab40772), Vimentin (1:1000, cat.no. ab92547), and N-cadherin (1:1000, cat.no. ab76011). After washed with TBST, the membranes were incubated with horseradish peroxidase (HRP)-labeled anti-rabbit secondary antibody (concentration 1: 3000) at room temperature for 1 h. The membranes were washed with TBST 3 times for 10 min each. Finally, Western blot special reagent (Invitrogen) was used for color imaging, and the gray value of each protein was analyzed by Image J (National Institutes of Health, Bethesda, MD, USA).

### Dual-luciferase reporter assay

All luciferase reporter vectors (SIRT3-wt, SIRT3-mut) were constructed by Promega Corporation (Promega, Madison, WI, USA). A549 and H1299 (4.5×10^4^) were seeded in 48-well plates and cultured to 70% confluence. Next, the cells were co-transfected with SIRT3-wt, SIRT3-mut and miR-224 mimics, inhibitors or negative controls using Lipofectamine 2000 (11668-027, Invitrogen Inc., Carlsbad, CA). Forty-eight h after transfection, the relative luciferase activity was analyzed by the Dual-GloLuciferase Assay System (Promega, Madison, WI, USA) according to the manufacturer's instructions. All experiments were performed in triplicate.

### Tumor formation in nude mice

A total of 40 BALB/nude mice (aged six weeks) was purchased from the animal center of Wuhan University. The NSCLC cells (A549 and H1299) were co-cultured with CAFs (transfected with miR-224 mimics) for 24 h. Then they were used to establish tumor formation models in the nude mice. The cell concentration was adjusted to 2×10^8^ ml^-1^. Next, 0.1 ml of cell suspension was injected into the subcutaneous armpit of the left forelimb of each nude mouse. Within five weeks after the injection, the survival rate, weight and survival status of the mice were monitored, and the tumor size and weight of the newly dead mice were measured. The animal study was approved by the Ethics Boards of Xiangyang Central Hospital.

### Data analysis

SPSS 17.0 statistical software (SPSS Inc., Chicago, IL, USA) was used for analysis. The measurement data were expressed as mean ± standard deviation (x ± s). A comparison between the two groups was tested by t-test. Data between multiple groups were compared by one-way variance analysis. The diversity was statistically significant with *P* <0.05.

### Ethical statement

Our study was approved by the Ethics Review Board of Xiangyang Central Hospital, Affiliated Hospital of Hubei University of Arts and Science.

### Data availability statement

The data sets used and analyzed during the current study are available from the corresponding author on reasonable request.
